# Sewage Farming and Sanitation

**Published:** 1896-06-27

**Authors:** 


### The Hospital

An Institutional Journal of

The Medical Sciences and Hospital Administration, with

AN EXTRA NURSING SUPPLEMENT.

Vol. XX.—No. 509.] [June 27, 1896.

### Sewage Farming and Sanitation.

A brief article published in The Hospital two or three weeks ago on the subject of meat and milk on sewage farms excited a good deal of attention in certain towns where sewage is disposed of by the farming system, and was freely commented upon in the local press. The comments and letters disclosed the fact that sewage farming may be so carried out as to be decidedly injurious to the surrounding neighbourhood, as well as being less scientific and less profitable than it need be.

The "cropping" of a sewage farm is perhaps the most important of all the operations carried on. There is one principle of cropping that should guide the decision of the crop producer in the selection of the use of every rood of land on his farm which is of first-rate importance. That principle is the selection for crops of those vegetables, plants, or shrubs which will take in the largest possible quantity of sewage whilst giving out in the form of exhalations the smallest possible quantity. This may be called "the law of cropping" on sewage farms. For the scientific application of this principle it is evident that some knowledge of botany is required. Now quick-growing plants will generally absorb sewage very rapidly. They will also exhale the odour of it with equal rapidity. Moreover, they themselves are the subjects of very rapid decomposition. Anyone who is practically familiar with the growing and decomposition of such crops as turnips, mangolds, marrows, and the like knows that this is exactly so. Obviously, then, such crops as these are relatively ill-fitted for extensive selection on sewage farms.

The English sewage farmers do not appear to be aware to any extent that there are varieties of shrubs and trees which might be of the greatest possible service in the utilisation of large sewage masses. So far as we are aware no experiments on an extensive scale have yet been made in England with forest trees. But it is highly probable that small plantations, of a few acres each, of eucalyptus trees, which it is well known produce admirable sanitary changes in the malarious regions of British Central Africa and elsewhere, would be of incalculable advantage in sewage farms of large size. At Birmingham, for example, at the present time, it is proposed to purchase an additional farm for the utilisation of sewage at a cost of nearly a quarter of a million sterling. If a farm of that acreage could be studded with some dozens of small eucalyptus forests each separate plantation would absorb immense quantities of sewage effluent, whilst the exhalations from the leaves instead of being injurious would help to purify the atmosphere of the whole farm and surrounding neighbourhood. Besides, how economical a "crop" would such a plantation be in the matter of labour! Once planted, the annual cost of keeping such forests in order would be almost nil. The immediate return would, of course, be nil also. But that would be quite a secondary point in the case of a corporation, whose primary business is not to make profit, but to get rid of its sewage in the easiest and most wholesome way. It is plain from these considerations that there is the widest possible scope for improvement in the very important matter of the cropping of sewage farms.

With respect to the feeding and management of cattle on farms of this order we suspect that little or no application of scientific principles is ever attempted. Milking cattle, fatting cattle, laying hens, pigs being fatted for the market, are all fed on the direct produce of the farms indiscriminately. But a little appreciation of the facts of elementary physiology would effect wonderfully improved results. For example, a bullock fatted on sewage produce, and killed directly from such produce, would have the flavour and quality of its flesh seriously deteriorated. But the same bullock might be reared on sewage produce and kept on such produce until within six months of the time it was designed for market. If, during the last six months, or even much less, of its life, it were fed on grass, or corn and hay and roots, which had not been produced by sewage agency, but on ordinary soil, then the sewage flavour and quality of its flesh would entirely disappear, and its beef would be of a quality similar to that of other bullocks of its age. That is to say, with a change of food of sufficient duration, physiology and experience tell us that we get a corresponding change of flavour and quality of beef. In like manner chickens designed immediately for the table should not be fed on sewage farm products; nor should hens which are laying eggs. But chickens intended to be kept for hens might properly be reared on sewage-grown products until within two months of the egg-laying period; and hens during their non-laying intervals might be kept on sewage-grown products also. The same rule applies to cows in milk and to cows not in milk, to "fatting" pigs and to "store" pigs, to pigeons, rabbits, and whatever sort of creature is reared on the farm for sale and profit.

In this article we have but glanced at a very important and at the same time very wide subject. As the population of Great Britain increases, whilst the area of the country remains, and cannot but remain, the same, the disposal of sewage may become the most serious and urgent of all the problems of medicine. If botany and physiology, with other sciences, will help us to solve this problem so as effectually to conserve health, the sooner our managers of sewage farms become botanists and physiologists the better will it be for us all.

